# First person state/psychological imagery modulates the orbital frontal gyrus and the putamen in individuals with depression

**DOI:** 10.3389/fpsyt.2025.1583482

**Published:** 2025-05-19

**Authors:** Barbara Tomasino, Maria Chiara Piani, Eleonora Maggioni, Carolina Bonivento, Serena D’Agostini, Matteo Balestrieri, Paolo Brambilla

**Affiliations:** ^1^ Scientific Institute, IRCCS E. Medea, Dipartimento/Unità Operativa Pasian di Prato, Udine, Italy; ^2^ Swiss Paraplegic Research, Nottwil, Switzerland; ^3^ Department of Electronics, Information and Bioengineering, Politecnico di Milano, Milan, Italy; ^4^ Department of Neurosciences and Mental Health, Fondazione IRCCS Ca’ Granda Ospedale Maggiore Policlinico, Milan, Italy; ^5^ Neuroradiology, Azienda Sanitaria Universitaria Friuli Centrale, ASU FC, Udine, Italy; ^6^ Psychiatry Unit, Department of Medicine, University of Udine, Udine, Italy; ^7^ Department of Pathophysiology and Transplantation, University of Milan, Milan, Italy

**Keywords:** state/psychological stimuli, emotion, imagery, depression, fMRI

## Abstract

**Background:**

Patients with depression appear to imagine scenes preferentially from a 3rd person perspective as if they were observers. Methods: By using functional magnetic resonance imaging we asked patients with major depressive disorder (19, MDD) and healthy controls (19, HC) to imagine 1st person perspective state/psychological (vs. action) scenes.

**Results:**

We found that the left superior frontal gyrus (orbital part) and the right putamen were differentially activated by the state/psychological (vs. action) imagery (vs. letter detection) in MDD individuals vs. HC.

**Discussion:**

We suggest that imagery for state/psychological events increased activation in areas involved in self-related processing and personal perspective changes.

## 1 Introduction

In psychopathology mental imagery is considered both a factor capable of maintaining affective symptomatology and a cognitive skill frequently used in psychological treatments (e.g., 1). Intrusive and unwanted imagery (e.g., 2) is present trans-diagnostically across major psychopathologies (e.g., 3–5). In particular, Holmes et al. (6) have examined mental imagery in depression, addressing its phenomenology, the putative altered mechanisms, and its potential therapeutic applications. Holmes et al. (6) argued that mental imagery alterations in depression involve both a hyperproduction of highly intrusive negative mental images (e.g., 2, 6–8), and an impoverishment of positive mental images (6, 8–12). In addition, depression is related to low mental imagery vividness (13, 14). Interestingly, an additional feature involves the perspective of imagination. Patients with depression appear to use 3rd person perspective imagery as if they were observers (6). In the case of autobiographical memory, 3rd person perspective imagery triggers a reduced emotional impact, as compared with 1st person perspective imagery (e.g., 15, 16). This is quite obvious, as 1st person’s perspective mental imagery triggers the simulation of the corresponding experience, an “as real” emotional experience (5). Contrarily, patients with depression tend to recall 3rd person perspective memories (e.g., 17–20). This strategy could represent a form of avoidance, especially when the content of emotions is negative (19) but also when it is positive (21–23).

While the behavioral pattern of mental imagery in depression has been extensively studied, the understanding of the functional brain correlates of mental emotion imagery in depressed individuals is less explored. Specifically, to the best of our knowledge, there is no functional Magnetic Resonance Imaging (fMRI) study addressing the brain activity underlying emotional imagery in individuals with depression. In one review (24) summarizing functional brain imaging studies of depression it was reported that many studies employed the resting-state paradigm. Out of the included task-based studies, paradigms only involved emotional processing, reward processing, and cognitive control. Similarly, in another review of fMRI studies in depression (25), no single study employing the emotion imagery paradigm was reported.

To fill this gap, we utilized a mental imagery fMRI paradigm in a group of 19 individuals with depression, comparing them to 19 healthy controls. The fMRI task has been previously employed with healthy young (26) and adult (27, 28) participants. Participants were prompted to create a 1st-person perspective mental image of the state/psychological verb’s content and then assess its pleasantness. On the same list of stimuli, participants performed a control task consisting of letter detection. As there is no fMRI study on state/psychological imagery in depressive disorders, we did not formulate hypotheses on which (if any) brain structures might show differential activations in depressed subjects when compared to healthy controls. In Tomasino et al. (27) we showed that the imagery task in healthy controls activated a network of areas involving the middle and superior occipital gyrus and parietal cortex bilaterally, the middle temporal gyrus bilaterally (extending to the right superior temporal gyrus), the left postcentral gyrus (extending to the supramarginal gyrus) and several clusters in the frontal cortex (the left superior frontal gyrus, the left precentral gyrus, the left middle frontal gyrus, the inferior frontal gyrus bilaterally and the right insula). Based on this network it can be hypothesized that activation in the parietal or frontal cortex, which are the most activated regions, may show differential activations in depressed subjects when compared to healthy controls. In addition, based on the available literature on mental imagery in depression cited above, we expected to find hyper-activation in areas related to first-person perspective imagery since patients with depression appear to imagine scenes preferentially from a third-person perspective, and our instructions forced them to imagine in a 1st person perspective.

## 2 Materials and methods

### 2.1 Subjects

A total of 38 participants were included in the study. The sample was composed of 19 individuals affected by major depressive disorders (MDD, mean age 42 ± 14.6, 16/19 females) and 19 healthy controls (HC, mean age 42.1 ± 15.3, 14/19 females). Exclusion criteria for HC were any diagnosis of psychiatric disorders, history of alcohol or substance abuse, head trauma, neurological or major medical illnesses, and history of psychiatric disorders in first-degree relatives. Exclusion criteria for patients included the presence of other comorbid axis I disorders, neurological or medical disorders with possible effect on brain development, history of traumatic head injury with loss of consciousness and alcohol or substance abuse. The research protocol was approved by the competent Research Ethical Committee of the Research Institute IRCCS E. Medea by the 2013 Fortaleza version of the Helsinki Declaration and subsequent amendments. All participants provided written informed consent to the study.

### 2.2 Psychopathological assessment

The clinical diagnoses were obtained using the Italian version of the Structured Clinical Interview for Axis I DSM-IV disorders (SCID-I) (29) and confirmed by the clinical evaluation of two expert psychiatrists. For the HC group, a brief non-patient version of the SCID-I was used to confirm the absence of psychiatric axis I disorders. Moreover, the psychopathological assessment included the 24-item Brief Psychiatric Rating Scale (BPRS) (30) and the Hamilton Anxiety and Depression Rating Scales (HARS and HDRS, respectively) (31, 32). The social, occupational, and psychological functioning were also assessed with the Global Assessment of Functioning scale (GAF) (33), while the cognitive performances were assessed by the Raven Matrices Test (RMT) (34). Handedness was determined with the Edinburgh Handedness Inventory (EHI) (35). Refer to **Table 1** for detailed information on the socio-demographic and clinical characteristics of the sample.

**Table 1:** Psychopathological assessment scoring.

	MDD	HC	MDD vs. HC (p-value)
**Age**	42.0 ± 14.6 y	42.1 ± 15.3 y	0.817
**Sex**	16 F, 3 M	14 F, 5 M	0.426
SCID-I A (*15 MDD, 19 HC)	Depression	No diagnosis	\
BPRS (*16 MDD, 17 HC)	32.2 ± 6.5	25.0 ± 1.8	<0.001
HDRS (*16 MDD, 17 HC)	7.7 ± 5.2	2.6 ± 4.0	0.004
HARS (*16 MDD, 17 HC)	9.3 ± 5.6	4.7 ± 6.2	0.034
GAF (*16 MDD, 17 HC)	73.9 ± 7.7	88.9 ± 3.4	<0.001
RMT (*14 MDD, 14 HC)			
**Total**	49.4 ± 6.4	46.7 ± 7.8	0.323
**Percentile**	93.6 ± 10.0	90.0 ± 11.7	
**Group**	Group 1 = 6; group 2 = 7; group 3 = 1	Group 1 = 7; group 2 = 5; group 3 = 2	
**IQ**	124.6 ± 7.0	122.4 ± 8.4	0.456
EHI (*16 MDD, 17 HC)	16 R, 0 L	14 R, 3 L	\
SES-ED (*16 MDD, 16 HC)	12.9 ± 6.1	11.1 ± 4.0	0.339
SES-OC (*16 MDD, 16 HC)	23.6 ± 7.3	26.3 ± 9.0	0.357
SES-TOT (*16 MDD, 16 HC)	37.0 ± 7.8	37.2 ± 12.3	0.965
Marital status (*17 MDD, 17 HC)	Stable relationship = 3; divorced = 5; married = 2; never married = 5; widow = 2	Stable relationship = 2; divorced = 2; married = 7; never married = 6; widow = 0	\
Race (*18 MDD, 17 HC)	Caucasian = 18	Caucasian = 17	\
Mother tongue (*18 MDD, 17 HC)	Italian = 17; other = 1	Italian = 17; other = 1	\
BMI (*17 MDD, 17 HC)	24.2 ± 4.0	24.3 ± 3.5	0.953
Cigarette smoke (*15 MDD, 17 HC)	No = 9; yes = 6	No =15; yes = 2	0.066
Alcohol abuse lifetime (*17 MDD, 17 HC)	No = 17; yes = 0	No = 16; yes = 1	0.310
Substance abuse lifetime (*17 MDD, 17 HC)	No = 17; yes = 0	No = 17; yes = 0	\
**Therapy**	*selective serotonin reuptake inhibitor antidepressants*	.	.

(*): number of individuals with measure available. Mean +/- standard deviation are reported for continuous measures. ANX, anxiety disorder; BMI, Body Mass Index; BPRS, brief psychiatric rating scale; DEP, depression; ED, education; EHI, Edinburgh Handedness Inventory; HARS, Hamilton anxiety rating scale; HC, healthy controls; HDRS, Hamilton depression rating scale; IQ, intelligence quotient; LT, lifetime; m, months; n, number; n, number; NOS, not otherwise specified; OC, occupation; RMT, Raven’s matrices test; SCID-I, structured clinical interview for axis I DSM-IV; SES, socio-economic status; TOT, total; w, with; w\o, without; y, years. Continuous variables were compared using T-tests, whereas categorical variables were compared using Chi-squared tests.

### 2.3 Experimental design

#### 2.3.1 Neuroimaging data acquisition

A 3T Philips Achieva scanner (Philips, Best, the Netherlands) equipped with an 8-channel head coil was used to acquire Magnetic Resonance Imaging (MRI) data. Participants’ head movements were limited by using restraining foam pads. The fMRI data (612 volumes) were acquired using a T2*-weighted gradient echo planar imaging (GE-EPI) sequence (repetition time (TR)= 2500 ms, echo time (TE)= 35 ms, flip angle= 90°, 30 axial slices with no gap, 128 × 128 in-plane matrix, voxel size= 1.79 mm × 1.79 mm × 3 mm). In addition, a 3D T1-weighted MPRAGE turbo field echo (TFE) SENSE image (TR= 8.2 ms, TE= 3.76 ms, 190 axial slices with no gap, 240 × 240 in-plane matrix, voxel size= 1 mm3, total scan duration= 8 minutes and 53 seconds) was collected and used for morphological referencing of the fMRI results. Visual stimuli were presented through MRI-compatible VisuaStim Digital goggles (Resonance Technology Inc., Northridge, CA, USA), and subjects’ responses were collected via an MRI-compatible hand Evoke Response Pad (Resonance Technology Inc., Northridge, CA, USA) for the index and middle fingers. All subjects utilized the right hand, which was the dominant one, to respond. Before the fMRI experiment, subjects were trained to perform the task outside the MR environment.

#### 2.3.2 fMRI task

The event-related fMRI experiment included a mental imagery (I) task and a letter detection (LD) task. Instructions lasted 5 s (“You will be asked to read a series of words silently and respond to a yes-no question by pressing the corresponding button”). In the I task subjects were explicitly asked to form a mental image of the verb content from a first-person perspective and to determine its pleasantness by answering the question, “Do you like it?”. In the LD task, subjects were explicitly asked to identify a target letter in the word by responding to the question “Search: Is the “S” present?”. Stimuli could be state/psychological verbs (Sta/Psy) or motor verbs (M) for a total of 144 trials. 64% of M and 58% of Sta/Psy trials included the letter “S.”

Stimuli lasted 4 s and were followed by variable inter-trial intervals, with a duration jittered from 3250 to 4000 ms with incremental steps of 250 ms. In addition, 36 null events (i.e., blank screens) perceived as a prolongation of their inter-trial period were randomly interspersed among the event trials to increase the power of estimating the fMRI blood oxygenation level-dependent (BOLD) response (36). To avoid any potential priming effect, trial sequences were pseudo-randomized in the two tasks.

The Sta/Psy and M stimuli were previously validated in a rating study (27). They significantly differed in motor relatedness (t(35)=132.7, p<0.001), imageability (t(35)=19.43, p<0.001), relatedness to a psychological state/emotion (t(35)=-40.24, p <.001), and written frequencies (t(35)=2.25, p<.05). By contrast, they were matched for familiarity (t(35)=-.95,p>.05, n.s.) and length (t(35)=.55, p >.05, n.s.). Regarding the distribution of the Sta/Psy stimuli in the affective space, arousal and valence were collected in a previous study (37) on a sample of three groups of healthy volunteers of different ages. Participants rated arousal and valence on a scale from 1 to 9 on the Self-Assessment Manikin (SAM) (38). For the arousal, they reported the level of arousal triggered by the action or feeling or state described by the verb (score 1-9, 1= very calm; 9= very aroused). As regards the emotional valence, they reported how happy the action or feeling or state described by the verb made them feel (score 1-9, 1= very happy; 9= very unhappy). For the 16-19-year-old participants, the Sta/Psy stimuli had a mean arousal of 4.4 ± 1.5 and a mean valence of 5.5 ± 2.1.

#### 2.3.3 fMRI data analyses

Matlab R2019a (The Mathworks, Inc.) and Statistical Parametric Mapping (SPM12) software (https://www.fil.ion.ucl.ac.uk/spm) (39), its Marsbar toolbox (40), Matlab in-house scripts were used for data analysis and functions from the Statistics and Machine Learning ToolboxTM.

For each subject, the first 6 dummy volumes were excluded from the analyses. To reduce head motion artifacts, we used a least squares approach and a rigid-body transformation to spatially realign the remaining 606 fMRI volumes to the first reference volume. We used 3 mm in translation and 3 degrees in rotation thresholds for exclusion. No participants exceeded this threshold. The mean of the resliced fMRI images was used as a reference for co-registration with the subject’s structural T1-weighted image, which was segmented into different tissue types and used for estimation of the deformation field for spatial normalization into the Montreal Neurological Institute (MNI) space. The realigned fMRI volumes was normalized to the MNI space, spatially resampled them to 2 mm × 2 mm × 2 mm, and smoothed them using a 3D Gaussian kernel filter with 6 mm Full Width at Half Maximum (FWHM).

A voxel-based General Linear Model (GLM) activation analysis was run. The design matrix had four experimental conditions: LD_M, LD_Sta/Psy, I_M, I_Sta/Psy, their time derivative as well as the regressor coding for fixations. The design matrix included the subjects’ movement parameters as confounding regressors. After GLM β coefficient estimation, a subject-level contrast map was extracted for the four experimental conditions using the corresponding β value map.

We entered each subject’s contrast maps in a second-level flexible factorial GLM analysis. The flexible factorial design included subjects, task (I vs. LD), stimulus (Sta/Psy vs. M), and group (MDD vs. HC) as factors, and age and sex as covariates in interaction with the group factor.

After estimating the GLM β coefficients, group effects on task-by-stimuli, task, and stimuli, fMRI activations were assessed through two-sided t-tests on the corresponding linear combination of β values by properly selecting both group and condition regressors. A multiple comparison correction was performed via a cluster-based family-wise error (cFWE) correction, using as voxel-wise primary threshold p=0.001 and as cluster-level threshold p=0.05. The anatomical interpretation of the fMRI results was performed using the SPM Anatomy toolbox (41).

#### 2.3.4 Task performance analyses

A GLM with the participants’ Reaction Times (RTs) and the percentage of the participants’ likability responses for I_Sta/Psy and I_M as dependent variables and diagnosis, age, and sex as independent variables was run. Group differences were extracted via two-sided t-tests on the corresponding β coefficients (p<0.05). Unfortunately, in the MDD group, the responses of six individuals were not recorded due to technical problems and could not be included in the performance analysis.

### 2.4 Results

#### 2.4.1 Behavioral performance

Both the linear GLM model and the GLM model considering the diagnosis in interaction with age and sex did not report significant differences in RTs or likeability scores between the groups (**Tables 2**, **3**).

**Table 2:** Behavioral results: mean response times (RTs) for HC and MDD.

	MDD*	HC	t-Stat (linear model; interaction model)	p (linear model; interaction model)
RTs_I	2258,08 ± 674,77	1325,66 ± 561,29	0,0235	0,9814
			0,8786	0,3877
RTs_LD	1987,69 ± 701,36	2023,17 ± 403,72	-0,1821	0,8567
			1,006	0,3237
RTs_ Sta/Psy	2055,38 ± 672,78	2094,44 ± 435,44	-0,1415	0,8885
			0,7229	0,4762
RTs_M	2190,38 ± 686,14	2254,39 ± 469,37	-0,0115	0,9909
			1,2167	0,2346

* Data available for 13 subjects.

HC, healthy controls; MDD, major depressive disorder; P, p-value; RT, response times; I, imagery; LD, letter detection; Sta/Psy, state/psychological; M, motor; ms, milliseconds; SD, standard deviation.

**Table 3:** Behavioral results: mean likability responses for HC and MDD.

Condition		HC	MDD *	HC vs. MDD	
				t-Stat (linear model; interaction model)	p (linear model; interaction model)
I_Sta/Psy	Mean %	44.7	43.2	0.2660 -1.5309	0.7922 0.1379
I_M	Mean %	53.8	53.0	-0.6460 -1.4220	0.5235 0.1670

* Data available for 13 subjects.

HC, healthy controls; MDD, major depressive disorder; P, p-value; RT, response times; I, imagery; LD, letter detection; Sta/Psy, state/psychological; M, motor; ms, milliseconds.

#### 2.4.2 fMRI activations

##### 2.4.2.1 Task-by-stimuli interaction

**Figure 1:**
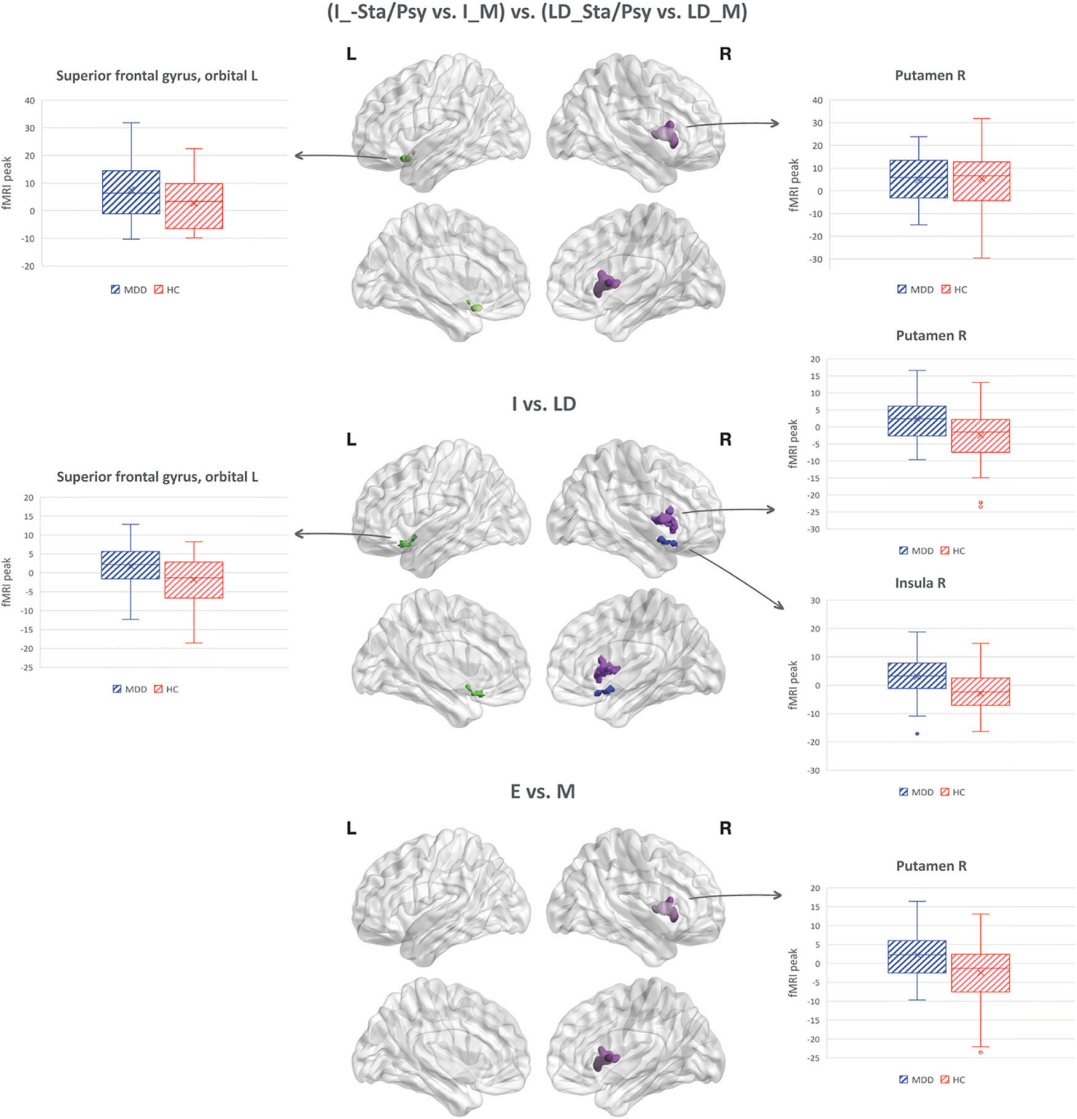
Task-by-stimuli interaction (p<0.05, cFWE corrected) along with the main effect of task and stimuli.

Increased BOLD response to the imagery (vs. letter detection) of emotion (vs. motor) stimuli [(I_Sta/Psy vs. I_M) vs. (LD_Sta/Psy vs. LD_M)] in MDD individuals compared to HC was found in the left superior frontal gyrus, orbital part and in the right putamen (see **Table 4**; **Figure 1**).

**Table 4:** Effect of MDD vs. HC difference on fMRI activations in the contrasts of interest.

Contrast	Effect	# voxels	x, y, z	AAL region	T	p (cFWE corrected)
[(I_-Sta/Psy vs. I_M) vs. (LD_Sta/Psy vs. LD_M)]	MDD > HC	87	-14, 14, -18	Sup frontal gyrus, orbital L	4.75	0.023
		300	18, 8, 6	Putamen, R	5.05	<0.001
I vs. LD	MDD > HC	73	28, 10, -14	Insula, R	5.44	0.048
		317	18, 8, 6	Putamen, R	5.12	<0.001
		89	-14, 14, -18	Sup frontal gyrus, orbital L	5.19	0.021
Sta/Psy vs. M	MDD > HC	244	18, 8, 6	Putamen, R	5.27	<0.001

I, imagery; LD, letter detection; E, emotional verbs; M, action verbs; [x, y, z], Montreal Neurological Institute coordinates (mm); AAL, Automated Anatomical Labeling; T, T-statistics; cFWE, cluster-based family-wise error; Sup, superior; L, left; R, right; MDD, major depressive disorder group; HC, healthy controls.

The correlation between the signal extracted from the frontal cluster (x=-14 y=14 z=-18) and the behavioral RTs data was not significant (I_E r(13)=.318, I_M r(13)=.411, LD_E r(13)=.262 and LD_M r(13)=.235), nor it was the correlation between the signal extracted from the putamen cluster (x=18 y=8 z=-6) and the behavioral RTs data (I_E r(13)= .696, I_M r(13)= .891, LD_E r(13)= -.603 and LD_M r(13)= .529).

The correlation between the signal extracted from the frontal cluster (x=-14 y=14 z=-18) and the HDRS data was not significant (r(16)=.335, p>.05, n.s.), nor it was the correlation between the signal extracted from the putamen cluster (x=18 y=8 z=-6) and the HDRS data (r(16)=-.151, p>.05, n.s).

##### 2.4.2.2 Main effect of task

<p>Increased BOLD response to I vs. LD in MDD individuals compared to HC was found in the right putamen, right insula, and left orbitofrontal cortex (see **Table 4**; **Figure 1**).

##### 2.4.2.3 Main effect of stimuli

Increased BOLD response to Sta/Psy vs. M stimuli in MDD individuals compared to HC was found in the right putamen (see **Table 4**; **Figure 1**).

### 2.5 Discussion

We investigated the neural correlates of emotion (vs. motor) -related imagery in a group of MDD compared to HC. At the behavioral level, MDD and HC performed similarly. However, we considered behavioral results to be poorly informative since they were available only for one-third of the MDD sample. This could, in principle, have affected the behavioral pattern. Thus, we decided not to comment on it further.

By contrast, the imaging dataset was complete. At the neural level, our main result is that imagery (vs. letter detection) of emotion (vs. motor) stimuli in MDD individuals compared to HC activated the left superior frontal gyrus, orbital part, and the right putamen.

As to the activation in the orbitofrontal cortex, neuroimaging literature focused on the neural basis of perspective-taking has extensively shown that this area is directly involved in self-awareness (42), self-related tasks and in perspective changes-related tasks (e.g., 43). For instance, processing sentences focused on the self (vs. another person) activates the orbitofrontal cortex (44–48), so this area has been related to the representation of self-related knowledge. Even more interestingly, for the present study, this area has been associated with tasks requiring changes in perspective, e.g., from a third-person perspective to a first-person perspective (49) and vice versa. We remind that our task instructions explicitly asked participants to imagine the scenes from a first-person perspective; we also reported in the introduction that, according to the literature, MDD appear to imagine scenes preferentially from a third-person perspective, as if they were observers (6). Therefore, the increased activation in the orbitofrontal cortex could be related to a continuous shift in the perspective the MDD participants assume during the task. Activation in the orbitofrontal cortex is found for imagining emotional scenes and not motor scenes. This would be consistent with the view that this area is preferentially involved in emotional perspective-taking, which consists of processing what the characters of a scenario are feeling, in contrast with cognitive perspective-taking, which consists of processing what the characters are thinking (50). Other studies on person perspective-related tasks reported activation in this area for both first-person perspective as compared to third-person perspective (51) and for third as compared to first-person perspective (49). Our results add further evidence showing that when instructed to imagine in a first-person perspective, activation in areas involved in self-other distinction is enhanced: this does not occur a-specifically whenever they imagined scenario, but only when they are engaged in emotion imagery.

As to the cluster of activation localized in the right putamen, we acknowledge that this result is not new, as it has been reported in a previous study of our group (28), in which we addressed the role of age and sex on mental imagery of emotion verbs. In detail, the same fMRI task has been used in the present and in the mentioned study. Activation in the right putamen was found to increase significantly in women, compared to men, for emotional imagery. In line with these results, in the present study, the sample of the MDD group included a majority of women (16 vs. 3 males). The putamen is involved in emotion processing as part of the striatum, a region that receives inputs from the amygdala (52–54), which in turn receives input from prefrontal areas. Our results further confirm the idea that the striatum is involved in imagery, emotion, and language processing (e.g., 55–58) and not simply in action planning and execution. The activation in the right putamen was also related to a general effect of stimuli, as the stimuli’ main contrast, performed on both the I and LD tasks, returned significant results in the right putamen, suggesting that the activation was related to processing Sta/Psy verbs (as compared with the other conditions) in MDD vs controls.

Unfortunately, no significant correlation was found among the behavioral scores, HDRS score and the imaging dataset. This could be due to the small sample size and further studies are planned to test this relation. Another possibility is that the signal extracted from the frontal and the putamen cluster does not reflect the time a participant took to imagine an emotional scene, but other imagery related variables like the imagery vividness for instance. Similarly, it is possible that the signal would better reflect a relation with 1^st^ vs 3^rd^ imagery vividness. Further studies are planned to test this relation.

### 2.6 Conclusion

Our study adds new and important neuroimaging evidence to the view that patients with depression tend to use a third-person perspective when imagining scenes and events (6). Likely they do so in order to diminish the emotional load (e.g., 15, 16), as the third-person perspective corresponds to imaging scenes as spectators. When forced to use a first-person perspective, as by the present fMRI paradigm, at the neural level, it is possible to detect an increase in activation in areas related to self-referential processing and perspective taking, likely because they are less used to doing so. Future fMRI studies in which we further investigate difference between first-person perspective and 3rd person perspective of fMRI data of MDD individuals are at the focus of future experiments. In the present study we limited our design to a 2x2 design with task (imagery, letter detection) and type of verbs (emotion/motor) as factors, to keep simpler the interpretation of putative interaction terms.

### 2.7 Limitation

Unfortunately, we do not have a behavioral counterpart of the fMRI pattern, as reaction times and likeability data were available only for 2/3 of the sample. This lack of behavioral information is a main limitation of the study. It leaves open the question of whether we would have detected (if the dataset were complete) some informative effects at the behavioral level, strengthening our fMRI results. A further limitation of the study is the lack of control in the fMRI analysis of the putative effect of clinical treatments, such as medication or psychotherapy. We acknowledge that in future studies, it is recommended that the impact of clinical treatment is taken into account when determining inclusion criteria. We acknowledge that the sample size of the two groups was small. Moreover, nearly one-third of the important behavioral data in the patient group was missing. Nonetheless we remark that the subjects in the study were in fact very homogeneous with accurate diagnoses and no comorbidities.

## Data Availability

The raw data supporting the conclusions of this article will be made available by the authors, without undue reservation.
